# Posterior Reversible Encephalopathy Syndrome in an Elderly Man: A Case Report

**DOI:** 10.7759/cureus.87987

**Published:** 2025-07-15

**Authors:** Carlos Nancassa, Tetiana Baiherych, Adelaide Figueiredo, Viktor Baiherych

**Affiliations:** 1 Internal Medicine, Hospital Distrital de Santarém, Santarém, PRT

**Keywords:** dynamic cerebral autoregulation, magnetic resonance imaging of the brain, posterior reversible encephalopathy syndrome (pres), systemic arterial hypertension, vasogenic edema

## Abstract

Posterior reversible encephalopathy syndrome (PRES) is a clinico-radiological entity characterized by the acute or subacute onset of neurological symptoms such as headache, seizures, visual disturbances, focal neurological deficits, and signs of encephalopathy. It is a syndrome with increasing recognition, largely due to the greater availability and utilization of neuroimaging techniques. We report the clinical case of a 74-year-old Caucasian male with a medical history of diabetes mellitus and obesity, whose main risk factor was arterial hypertension. He was admitted to the emergency department presenting with headache, mental confusion, speech disturbance, and decreased visual acuity, with one day of symptom progression. On admission, he was hypertensive (160/70 mmHg), somnolent but easily arousable, with disorganized speech and a Glasgow Coma Scale score of 12. Initial laboratory investigations showed no significant abnormalities. Cranial computed tomography revealed bilateral hypodense areas in the temporo-occipital regions, more pronounced on the left, with additional involvement of the anterior temporal and parietal lobes. Brain magnetic resonance imaging confirmed the presence of vasogenic edema, consistent with PRES. The patient remained hospitalized for clinical monitoring and blood pressure control, as well as further diagnostic workup. He showed marked clinical improvement, with complete resolution of symptoms within one week. Through this case report, we seek to underscore the importance for clinicians of considering PRES in the differential diagnosis of acute neurological presentations, even in patients without severe arterial hypertension or other commonly associated risk factors.

## Introduction

Posterior reversible encephalopathy syndrome (PRES) is a clinico-radiological entity first described in 1996 by Hinchey et al. in a series of 15 patients presenting with acute and subacute neurological symptoms, including headache, seizures, visual disturbances, focal neurological deficits, and signs of encephalopathy [1-4]. Although the incidence of PRES in the general population remains unknown, it has been reported in selected patient cohorts [3,5-8]. The most commonly described risk factor is the abrupt elevation of blood pressure [4]. Other predisposing factors include pre-eclampsia/eclampsia, autoimmune, hematological or infectious diseases, bone marrow or solid organ transplantation, renal failure, immunosuppressive or chemotherapeutic agents, and hydroelectrolytic disturbances, among others [2-12]. Diagnosis is based on the correlation between clinical and radiological findings in the presence of known risk factors [2]. Brain computed tomography (CT) may be useful as an initial examination, revealing hypodense areas in vulnerable regions, but diagnostic confirmation relies on brain magnetic resonance imaging (MRI) [3-7,12-15]. The parieto-occipital regions are involved in approximately 70% of cases, followed by frontal and temporal lobes [3-4,7]. The primary treatment goal is to address the underlying cause, including gradual blood pressure reduction, administration of antiepileptic drugs or sedation, discontinuation or replacement of triggering medications, correction of electrolyte disturbances with appropriate hydration, and prompt delivery in pregnant patients [4-5,7]. Secondary complications may include malignant PRES, large ischemic infarction, intracranial hemorrhage with mass effect, obliteration of basal cisterns, transtentorial, tonsillar, or uncal herniation, and status epilepticus [5,8]. Although the prognosis is favorable in most patients (75-90%) [2-5,9], with symptom resolution typically within two weeks, the term “reversible” is not always appropriate. Irreversible cases with varying degrees of functional impairment have been reported, with mortality rates ranging from 6% to 19% [2-5,16]. Prognosis depends on the underlying cause, early diagnosis, appropriate treatment, and imaging features [3,17].

## Case presentation

A 74-year-old Caucasian male with a medical history of non-insulin-dependent diabetes mellitus and obesity. Arterial hypertension was identified as a significant risk factor. His regular medications included metformin and a combination of perindopril with amlodipine. He was admitted to the emergency department presenting with headache, mental confusion, speech disturbances, and decreased visual acuity, with one day of symptom evolution. On admission, he was hemodynamically stable, with a blood pressure of 160/70 mmHg and a heart rate of 75 bpm. Neurologically, the patient was somnolent but easily arousable, with disorganized speech and a Glasgow Coma Scale score of 12. Cranial nerve assessment revealed poor cooperation with confrontation visual field testing, although he was able to count fingers at a distance of three meters. No focal neurological deficits were identified. The remaining physical examination did not show significant abnormalities. Laboratory evaluation revealed borderline magnesium levels (1.7 mg/dL) and preserved renal function (urea: 27 mg/dL; creatinine: 0.7 mg/dL), with an erythrocyte sedimentation rate (ESR) of 10 mm. Other laboratory results, including complete blood count, coagulation panel, and biochemistry (electrolytes, serum aminotransferases, gamma-glutamyl transferase, alkaline phosphatase, lactate dehydrogenase, albumin, total proteins, bilirubins, thyroid-stimulating hormone, free thyroxine, and creatine kinase), were within normal limits. Immunological tests (antinuclear antibody (ANA) and antineutrophil cytoplasmic antibodies (ANCA)), as well as serologies for human immunodeficiency virus types 1 and 2, hepatitis B, and hepatitis C, were performed to exclude other potential causes of PRES, all of which were negative (Table 1).

**Table 1:** Patient’s laboratory assessment

Parameter	Results	Reference values
Hemoglobin	13.7 g/dL	13-17 g/dL
Leukocytes	4.3 × 10^9^/L	4.0-10.0 × 10^9^/L
Neutrophils	1.8%	2.0-7.0%
Lymphocytes	1.8%	1.0-3.0%
Monocytes	0.4%	0.2-1.0%
Eosinophils	0.3%	0.0-0.5%
Basophils	0.1%	0.0-0.1%
Platelets	176 × 10^9^/L	150-410 × 10^9^/L
Hemosedimentation rate	10 mm/hour	<30 mm/hour
C-reactive protein (CRP)	0.34 mg/dL	<0.10 mg/dL
Prothrombin time (PT)	12.19 seconds	9.9-12.3 seconds
Activated partial thromboplastin time (aPTT)	25.60 seconds	25.0-35.0 seconds
Creatinine	0.7 mg/dL	0.7-1.3 mg/dL
Urea	27 mg/dL	18.0-55.0 mg/dL
Sodium	141 mmol/L	135-145 mmol/L
Potassium	4.0 mmol/L	3.5-5.1 mmol/L
Magnesium	1.7 mg/dL	1.7-2.6 mg/dL
Aspartate aminotransferase (AST)	16 U/L	5-34 U/L
Alanine aminotransferase (ALT)	19 U/L	<55 U/L
Alkaline phosphatase	60 U/L	40-150 U/L
Gamma-glutamyl transferase	52 U/L	<55 U/L
Total bilirubin	1.1 mg/dL	0.2-1.2 mg/dL
Lactate dehydrogenase (LDH)	134 U/L	125-220 U/L
Albumin	4.5 g/dL	3.2-4.6 g/dL
Total protein	7.1 g/dL	6.2-8.1 g/dL
Thyroid-stimulating hormone (TSH)	2.29 mUI/L	0.35-4.94 mUI/L
Free thyroxine (FT4)	0.99 ng/dL	0.70-1.48 ng/dL
Creatine kinase (CK)	184 U/L	30-200 U/L
Antinuclear antibodies (ANA)	1:40 titer	Titer < 1:160
Perinuclear antineutrophil cytoplasmic antibodies (pANCA)	5 U/mL	-
HIV types 1 and 2 (antibody + antigen P24)	Negative	-
Hepatitis B virus (HBV): Hepatitis B surface antigen (HBsAg)	Negative	-
HBV: Anti-hepatitis B surface (Hbs)	<2 UI/L	>10 IU/L positive
Hepatitis C virus (HCV): Anti-HBc total and anti-HCV	Negative	-

**Figure 1:**
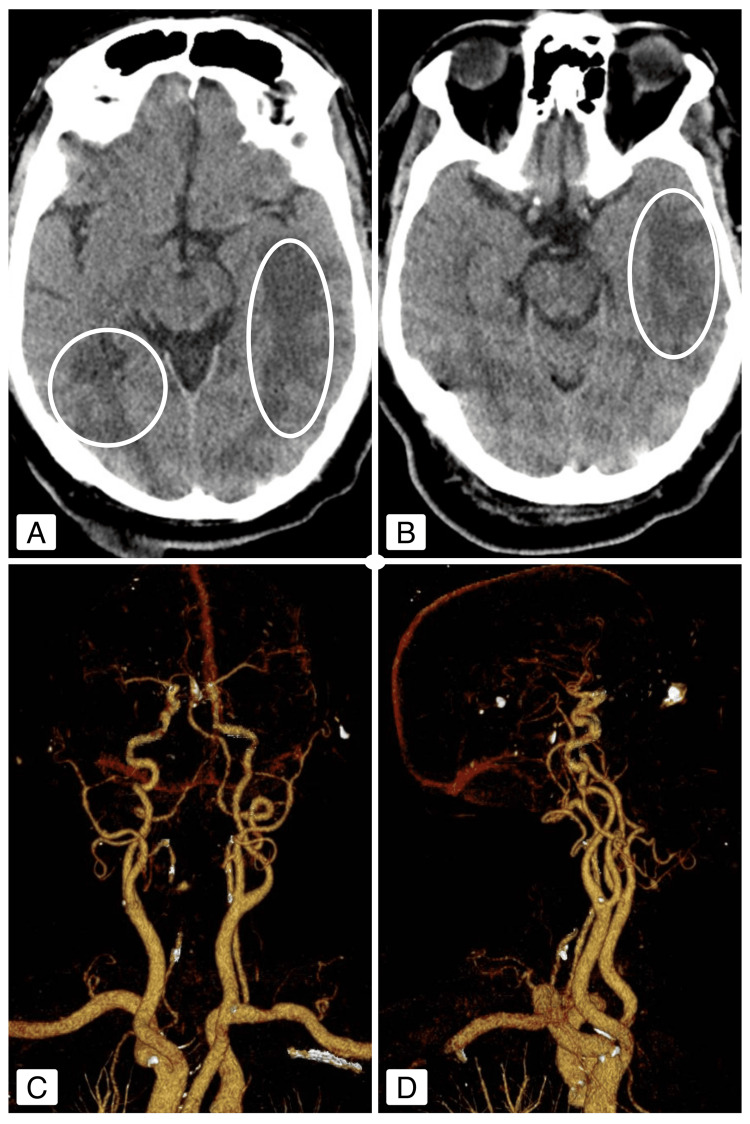
(A-B) Brain computed tomography (CT) showing bilateral hypodense areas in the temporo-occipital regions, more extensive on the left, with additional involvement of the anterior temporal and parietal lobes (circles highlighting the areas of lesion). (C-D) Computed tomography angiography (CTA) of the cervical and intracranial vessels with three-dimensional (3D) reconstruction, showing no significant stenosis or occlusion of the visualized arteries.

For diagnostic clarification, brain CT and CT angiography of the neck and intracranial vessels (Figure 1) were performed, revealing bilateral temporo-occipital areas of vasogenic edema, more pronounced on the left, also involving the anterior temporal and parietal lobes. There was associated sulcal effacement and ventricular system deformation, findings consistent with a possible diagnosis of PRES. No early signs of ischemia were identified, and no significant stenoses or occlusions of the major cervical or intracranial arteries were detected.

The patient remained hospitalized in the Observation Unit for clinical monitoring and blood pressure surveillance. There was marked improvement in the disorientation, with no significant blood pressure fluctuations and no requirement for intravenous antihypertensive therapy. Blood pressure was controlled with a dual combination of perindopril and amlodipine. The patient remained neurologically and hemodynamically stable, with no complications warranting admission to the intensive care unit.

**Figure 2:**
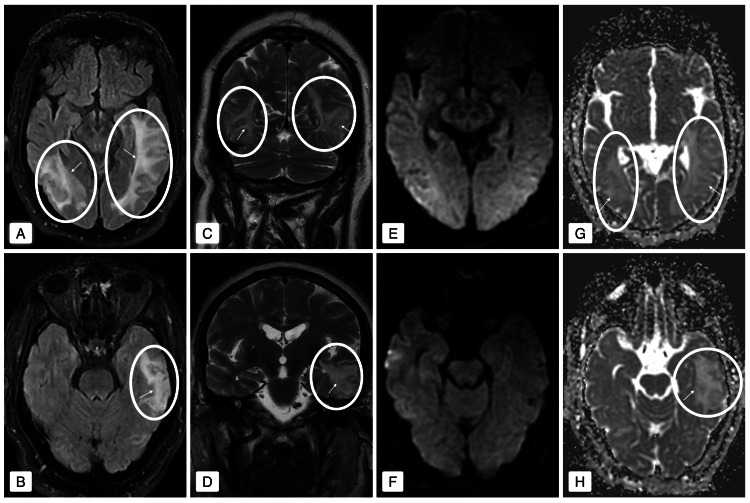
Brain magnetic resonance imaging (MRI). (A-D) Axial FLAIR and coronal T2-weighted images demonstrate subcortical hyperintensities with a digitiform pattern, predominantly affecting the occipital lobes and the left temporal lobe. (E-H) Diffusion-weighted imaging (DWI) shows no signal intensity abnormalities; however, the apparent diffusion coefficient (ADC) maps reveal elevated values, confirming the presence of vasogenic edema (circles and arrows highlight the areas of lesion).

He was subsequently transferred to the Internal Medicine Ward, where a brain MRI was performed (Figure 2). The MRI confirmed vasogenic edema, revealing, on fluid-attenuated inversion recovery (FLAIR) sequence, marked subcortical hyperintensities with digitiform morphology, predominantly in the occipital lobes and the left temporal lobe - findings consistent with PRES.

During hospitalization, the patient was re-evaluated by a neurologist, with no new findings, and showed progressive clinical improvement by the seventh day of admission. Potential ophthalmological complications associated with PRES were ruled out following ophthalmological evaluation, which documented a visual acuity of 9/10 in both eyes, an intraocular pressure of 16 mmHg bilaterally, normal ocular biomicroscopy, and a fundoscopic examination without pathological findings.

The patient was discharged on the 12th day of hospitalization following complete clinical resolution and achievement of target blood pressure levels (130/80 mmHg). Follow-up was maintained in the Internal Medicine Outpatient Clinic during the first six months due to the risk of PRES recurrence.

## Discussion

Hypertension with failure of cerebral autoregulation is believed to play a central role in the development of PRES [1-2,5-17]. In response to fluctuations in systemic blood pressure, cerebrovascular autoregulation preserves cerebral blood flow by inducing vasodilation during hypotension and vasoconstriction in cases of systemic hypertension. A sudden and marked increase in blood pressure may exceed the capacity of cerebral autoregulation (mean arterial pressure >150-160 mmHg), leading to hyperperfusion and vasogenic edema [2,15]. Furthermore, the rate of blood pressure elevation or the presence of abrupt fluctuations appears to carry a higher risk for the development of PRES than the absolute blood pressure value itself [15]. In our patient, we hypothesize that hypertensive fluctuations were the main predisposing factor, since blood pressure levels at admission were not markedly elevated. It is important to note that approximately 30% of PRES cases occur with normal or only mildly elevated blood pressure levels [2,15].

In addition to arterial hypertension, other pathophysiological hypotheses have been proposed to explain the development of PRES in different clinical contexts. Among them, endothelial dysfunction secondary to the release of endogenous or exogenous cytokines and toxins, seen in settings such as sepsis, eclampsia, immunosuppressive therapies, or autoimmune diseases, may lead to disruption of the blood-brain barrier with subsequent vascular leakage [2,4,10-11], as well as focal vasospasm leading to local hypoperfusion and ischemia with resultant edema [10-12]. These last two hypotheses seem less likely in the present case, considering the patient’s medical history and the diagnostic investigation during hospitalization, which did not reveal any other plausible etiologies.

The exact incidence of PRES in the general population remains unknown. However, data from specific adult cohorts indicate rates ranging from 2.7-25% after bone marrow transplantation, 0.4-6% following solid organ transplantation, 0.84% in patients with end-stage renal disease, and 0.69% in cases of systemic lupus erythematosus [3,5-8]. In the pediatric population, the incidence of PRES varies between 0.04% and 0.4% in intensive care units [5,8]. The most frequently associated triggering factors include preeclampsia/eclampsia, arterial hypertension, renal failure, cytotoxic agents, and autoimmune diseases [2-3,5,8,12].

Typical clinical manifestations of PRES include headache, altered mental status (ranging from somnolence to stupor), seizures, focal neurological deficits, and visual disturbances such as blurred vision, hemianopia, visual neglect, and, in more severe cases, cortical blindness [1,2,5-7]. These symptoms usually develop over the course of hours to a few days, with a tendency to progressively resolve following correction of the precipitating factors and appropriate treatment of the underlying cause [11-14]. Non-convulsive status epilepticus should be considered as a possible presentation of PRES in comatose patients, although it is not a common manifestation [4]. In the case presented, the patient exhibited symptoms consistent with PRES without complications, and the brain MRI findings were suggestive of a moderate form of the syndrome. The diagnosis was established through clinico-radiological correlation, in association with a known risk factor (arterial hypertension).

CT may be useful as an initial imaging modality, revealing hypodense areas in typically affected regions. However, diagnostic confirmation is achieved through brain MRI [3-7,12]. T2-weighted and FLAIR sequences reveal cortical and subcortical hyperintensities corresponding to vasogenic edema [12]. The differentiation between vasogenic and cytotoxic edema is accomplished using diffusion-weighted imaging (DWI) and apparent diffusion coefficient (ADC) maps [12-14]. Vasogenic edema presents as isointense or hypointense on DWI, whereas cytotoxic edema appears hyperintense. On ADC maps, vasogenic edema shows preserved or increased values, whereas cytotoxic edema is associated with decreased values [7,12,15]. The parieto-occipital regions are involved in approximately 70% of cases, followed by the frontal and temporal lobes [3-4]. The posterior predominance may be attributed to the lower sympathetic innervation of these areas compared to the anterior circulation, resulting in greater vulnerability to blood pressure changes [2,7]. Involvement of other regions such as the brainstem, basal ganglia, internal capsule (posterior limb), cerebellum, and periventricular regions is less common [3-4,6-7,12-15]. Spinal cord involvement is rare [3].

The differential diagnosis of PRES includes encephalitis (viral, autoimmune, or paraneoplastic), demyelinating diseases, central nervous system vasculitis, osmotic demyelination syndrome (central pontine and extrapontine), gliomatosis cerebri, hypoxic-ischemic encephalopathy, hepatic or toxic encephalopathy, progressive multifocal leukoencephalopathy, status epilepticus, acute ischemic stroke, dural venous sinus thrombosis, reversible cerebral vasoconstriction syndrome, acute hepatic encephalopathy, and SMART (Stroke-Like Migraine Attacks After Radiation Therapy) syndrome, among others [5,7,10,14-15].

Regarding therapeutic management, there are no specific randomized clinical trials addressing the treatment of PRES, owing to its relative rarity and acute presentation [2-4,8,15]. The primary goal is to treat the underlying cause, including gradual blood pressure control (with a reduction of 20-25% within the first hours). First-line agents include nicardipine, labetalol, and nimodipine; whereas second-line agents include sodium nitroprusside, hydralazine, and diazoxide [4-5,7]. Additional measures include the use of antiepileptic drugs, sedation, discontinuation or substitution of causative medications, correction of electrolyte disturbances with hydration, and immediate delivery in pregnant women [4-5,7]. Neuroimaging follow-up is essential to monitor therapeutic response and exclude differential diagnoses [4]. Approximately 70% of patients require admission to an intensive care unit, including invasive mechanical ventilation, transfusion of blood products, corticosteroid therapy for autoimmune diseases, intracranial pressure monitoring, cerebrospinal fluid drainage, and, in some cases, decompressive craniectomy [4,7]. Common indications for ICU transfer include encephalopathy, seizures, and status epilepticus [7]. Secondary complications such as malignant PRES, massive ischemic stroke, intracranial hemorrhage with mass effect, transtentorial, uncal, or tonsillar herniation, and status epilepticus may lead to permanent neurological sequelae and death [5,8].

Although the prognosis is favorable in most cases (75-90%) [2-5,9], with clinical improvement usually observed within the first two weeks, the term “reversible” is not always accurate, as irreversible forms with varying degrees of functional impairment have been reported, and estimated mortality ranges from 6-19% [2-5,16]. Factors such as the underlying etiology, early diagnosis, therapeutic intervention, and imaging findings directly influence outcomes [3,17]. Recurrences have been described in patients with predisposing conditions such as sickle cell disease, autoimmune disorders, hypertensive crises, renal failure, and multiple organ dysfunction [3-5].

## Conclusions

This case highlights the importance of considering PRES in the differential diagnosis of acute neurological presentations, especially in patients presenting with encephalopathy and visual disturbances, even in the absence of severe arterial hypertension or other commonly associated risk factors. Early diagnosis is essential to prevent morbidity, mortality, and neurological sequelae. Strict blood pressure control and treatment of the underlying condition are crucial for rapid recovery and a favorable prognosis.
